# Despotism and Risk of Infanticide Influence Grizzly Bear Den-Site Selection

**DOI:** 10.1371/journal.pone.0024133

**Published:** 2011-09-14

**Authors:** Nathan S. Libal, Jerrold L. Belant, Bruce D. Leopold, Guiming Wang, Patricia A. Owen

**Affiliations:** 1 Carnivore Ecology Laboratory, Forest and Wildlife Research Center, Mississippi State University, Mississippi State, Mississippi, United States of America; 2 Department of Wildlife, Fisheries, and Aquaculture, Mississippi State University, Mississippi State, Mississippi, United States of America; 3 Denali National Park and Preserve, Denali Park, Alaska, United States of America; University of Western Ontario, Canada

## Abstract

Given documented social dominance and intraspecific predation in bear populations, the ideal despotic distribution model and sex hypothesis of sexual segregation predict adult female grizzly bears (*Ursus arctos*) will avoid areas occupied by adult males to reduce risk of infanticide. Under ideal despotic distribution, juveniles should similarly avoid adult males to reduce predation risk. Den-site selection and use is an important component of grizzly bear ecology and may be influenced by multiple factors, including risk from conspecifics. To test the role of predation risk and the sex hypothesis of sexual segregation, we compared adult female (*n* = 142), adult male (*n* = 36), and juvenile (*n* = 35) den locations in Denali National Park and Preserve, Alaska, USA. We measured elevation, aspect, slope, and dominant land cover for each den site, and used maximum entropy modeling to determine which variables best predicted den sites. We identified the global model as the best-fitting model for adult female (area under curve (AUC) = 0.926) and elevation as the best predictive variable for adult male (AUC = 0.880) den sites. The model containing land cover and elevation best-predicted juvenile (AUC = 0.841) den sites. Adult females spatially segregated from adult males, with dens characterized by higher elevations (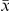 = 1,412 m, *SE* = 52) and steeper slopes (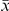 = 21.9°, *SE* = 1.1) than adult male (elevation: 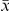 = 1,209 m, *SE* = 76; slope: 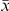 = 15.6°, *SE* = 1.9) den sites. Juveniles used a broad range of landscape attributes but did not avoid adult male denning areas. Observed spatial segregation by adult females supports the sex hypothesis of sexual segregation and we suggest is a mechanism to reduce risk of infanticide. Den site selection of adult males is likely related to distribution of food resources during spring.

## Introduction

Animal distribution theory has two pervasive models: the ideal free distribution and ideal despotic distribution models. The ideal free distribution model applies to non-territorial animals and states individuals are distributed proportionately to resources available [1]. Under this model, individuals assess the quality of available habitats and move unhindered among habitat units to select those considered best [1]. The ideal despotic distribution model applies to territorial animals, with dominant individuals displacing subordinates from higher quality habitats [2]. Subordinates' selection of habitat is therefore constrained by the distribution and behavior of dominant individuals [2]. This displacement in part forms an animal's realized niche [3]. Evidence for ideal despotic distribution has been demonstrated across a broad range of taxa [4]–[7].

Risk of predation and infanticide has long been hypothesized to influence behavior and resource selection in animals [8]–[12]. Though studies of evolutionary responses to risk have largely focused on predator/prey systems, evidence of these responses to conspecifics has also been found [10]–[23]. For example, dispersal in juvenile field voles (*Microtus agrestis*), cougars (*Puma concolor*), and Iberian lynx (*Lynx pardinus*) apparently serves in part to reduce risk from conspecifics [13], [17], [22]. Habitat selection by juvenile Atlantic cod (*Gadus morhua*) and seal salamanders (*Desmognathus monticola*) is also affected by risk from larger conspecifics [10], [16]. To reduce infanticide risk by unfamiliar males, many primate species have evolved permanent male-female associations [19]. Sexual segregation is another method by which mammal species with male-biased size dimorphism appear to reduce risk from conspecifics [11], [12], [14]. In some cases, female African lions (*Panthera leo*) and their young abandon prides and become temporarily nomadic when a new male has taken over, thus avoiding infanticide by the new dominant male [15]. Similarly, female alpine marmots (*Marmota marmota*) and their young may shift territories when new males encroach [18]. Resource partitioning between adult male and adult female cougars with young may also reduce risk of infanticide through sexual segregation [21], [23]. Though empirical evidence for decreased infanticide events in segregating individuals is lacking, segregated female alpine marmots did successfully wean young [18], [20]. Sexual segregation by mature females to protect young from immigrant adult males is known as the sex hypothesis of sexual segregation [14], [24].

Sexual size dimorphism is common in many species, including those with polygynous and promiscuous breeding strategies and has been demonstrated to result in sexual segregation [12], [23], [24]. In bear (*Ursus* spp.) populations, adult males are physically larger and dominant over other sex/age classes [12]. Increased body size in male bears is also positively associated with within-group dominance and increased breeding opportunities [25], [26], [27]. Consumption of abundant, highly digestible food increases grizzly bear (*U. arctos*) size and condition [28], [29]. Thus, individuals with access to high quality food sources benefit from increased body size, and therefore improved fitness [28].

Intraspecific predation has been observed in bear populations, in which adult males have killed juveniles (independent, non-breeding individuals) and adult females [30]–[33]. Although mechanisms driving intraspecific predation in bears are not completely understood, intraspecific aggression and population regulation may be involved [30]–[32], [34]. To reduce predation risk, juvenile grizzly bears may spatially and temporally segregate from dominant, non-kin adult males [35]–[37]. Sexual segregation has also been observed in grizzly bear populations, with mature females avoiding male-occupied habitats, potentially to reduce risk of infanticide [24]. Infanticide of unrelated young may provide a reproductive advantage for the infanticidal male, as females without young may be brought into estrous earlier and bred by the infanticidal male [38], [39]. In Alaska, spatial distribution of bears near salmon (*Oncorhynchus* spp.) streams appears driven by adult male bear presence, with adult females with young avoiding streams frequented by adult males [12]. Seasonal range size of adult females also appears influenced by risk of infanticide in Scandinavia, where oestrous females occupy a larger area during the mating season while females with cubs select small ranges to avoid males [11].

Den selection and use is an important component of bear ecology. Bear hibernation is generally attributed to limited food resources during winter [40], when bears reduce energetic costs by reducing metabolic rates [41]. Den sites may also provide thermal advantages and likely provide a secure location for parturition [42], [43]. Energetic demands of hibernation result in substantial body mass loss [29], [44]; therefore, it would be advantageous for all bears to locate dens near areas where food is likely to be abundant shortly after den emergence in spring. However, spatial segregation between sex/age groups of bears has been reported [45], [47]. Juveniles and adult females, particularly females with dependent young, may be more vulnerable to predation and infanticide by male bears during hibernation [30], [45]. Consequently, risk from conspecifics may influence den-site selection in grizzly bears. To reduce predation risk, juveniles may spatially or temporally segregate from adult males during the denning season. As infanticide is maladaptive to adult female grizzly bears, they may also modify timing and location of den sites to avoid males, as suggested for polar bears (*U. maritimus*; [46]).

To avoid detection by adult male grizzly bears, juveniles and adult females may den at higher elevations [45], [47], arrive at these sites earlier [47]–[49] and leave the denning area later [47]–[49] than adult male bears. Our objective was to test if spatial distribution of grizzly bear den sites supports the ideal despotic distribution model. We also tested whether the sex hypothesis of sexual segregation [14], under the umbrella of ideal despotic distribution theory, further explains den-site selection of adult female grizzly bears. We predicted that in order to reduce risk of infanticide and predation, adult females and juveniles spatially segregate from adult males by choosing den sites at higher elevations than adult males. We further predicted that adult females segregate to a greater extent than juveniles in order to protect their reproductive investment from potentially infanticidal males.

## Methods

### Ethics Statement

Ethics approval was not required at the time animal capture occurred. However, animal capture and handling procedures followed guidelines established by the American Veterinary Medical Association and American Society of Mammalogists.

### Study Area

The study area encompassed about 7,068 km^2^ of Denali National Park and Preserve (63°3′24.63″N 150°49′19.44″W). Temperatures vary depending on elevation and season; reaching 32°C in summer and dropping to −47°C in winter [50]. Study area elevations range from 152–4,116 m. The region lies partially in the rain shadow of Denali and receives less precipitation than areas south of the mountain. Still, winter snow pack reaches depths of about 200 cm [50]. White spruce (*Picea glauca*), birch (*Betula* spp.), and aspen (*Populus* spp.) are common tree species from valley bottoms to about 700 m. Willow (*Salix* spp.) and alder (*Alnus* spp.) are common from about 500 m to treeline (500–1,300 m). Mountain avens (*Dryas* spp.) mats and lichens are abundant in tundra (≥1,000 m). Several forms of disturbance are common in the study area, including the Muldrow Glacier, ice action (erosion due to ice flows during spring break up) along waterways, and wildfires at lower elevations [50]. Population trends for the resident grizzly bears were summarized previously [51] and are generally stable, with an estimated lambda = 0.9963, *SE* = 0.0166. The estimated mean litter size for this population is 2.03 cubs/litter, and the estimated annual reproductive rate = 0.35, *SE* = 0.04. Cub and yearling survival rates are relatively low at about 0.34, *SE* = 0.04 and 0.60, *SE* = 0.07 respectively. Young generally stay with the mother for 2 years before dispersing. Subadult and adult survival rates are high at approximately 0.96, *SE* = 0.04 and 0.96, *SE* = 0.01 respectively. In addition to grizzly bears, the area supports populations of black bears (*U. americanus*), wolves (*Canis lupus*), caribou (*Rangifer tarandus*), and moose (*Alces alces*) [52]. Though there are few human settlements in the park, Denali National Park and Preserve receives 350,000–460,000 visitors annually (National Park Service 2010).

### Data Collection

Between 1990–1998, grizzly bears were captured by Denali National Park and Preserve staff using aerial darting and fitted with very high frequency (VHF) radiocollars [53]. Bear ages were estimated by counting cementum annuli from an upper premolar (Matson's Laboratory, Milltown, Montana, USA) [54]. Bears were classified as adult female (≥5 years old), adult male (≥5 years old), or juvenile (≤4 years old). We used age four as the cut-off for juveniles based on later reproduction for northern grizzly bears [3], [55]. Den sites were located between September and May each winter using aerial telemetry, plotted on United States Geological Survey (USGS) 7.5 minute topographic maps, and converted to Universal Transverse Mercator grid coordinates.

Four landscape variables were used to classify the exact den locations (single pixel): land cover, elevation, slope, and aspect. Land cover was the dominant vegetation community or other surface cover type as classified by the Earth Satellite Corporation and the National Park Service (DENA Land Cover Mapping Project, ∼48 m^2^ pixel size, 2001). Several land cover types were combined based on vegetative similarities: open woodland spruce with open woodland/stunted spruce, broadleaf with mixed forest, alder shrub with willow shrub, low shrub/birch/willow with low shrub/sedge, dwarf shrub with dwarf shrub/rock, wet herbaceous with aquatic herbaceous, sparse vegetation with bare ground, and silty water with clear water for 12 cover types (Table 1). Elevation, slope, and aspect were obtained for the pixel containing each den site using a USGS digital elevation model (US GeoData – Alaska, ∼48 m^2^ pixel size, 2010). Elevation of each pixel was classified to the nearest meter and slope was classified as 0–90°. Aspect of each pixel was converted from degrees to a cardinal direction (north = 316–45°; east = 46–135°; south = 136–225°; west = 226–315°; or flat) [56]. All landscape data was extracted using ArcMap 3.9 (Environmental Systems Research Institute, Redlands, California, USA). From initial evaluations of habitat patch size, estimated maximum location error, and pixel resolution, accuracy of resource metrics extracted was appropriate for analyses [57], [58].

**Table 1:** Covertypes used to classify adult female, adult male, and juvenile grizzly bear den sites, Denali National Park and Preserve, Alaska, USA 1990–1998.

Covertype	Description
Dense conifer forest	Dense canopy forest dominated by spruce
Open woodland spruce	Open canopy forest dominated by spruce
Broadleaf/mixed forest	Open or dense canopy forest with multiple species
Alder/willow shrub	Shrub community dominated by alder and willow
Closed low birch shrub	Dense shrub community dominated by birch
Low shrub/birch/willow/sedge	Open or dense shrub community with multiple species
Dwarf shrub	Open or dense shrub community with smaller plants
Dry herbaceous	Open herbaceous community associated with drier sites
Wet herbaceous	Open herbaceous community associated with wet sites
Sparse vegetation	Characterized by mixture of bare soil, rock, and herbaceous plants
Snow/ice	Characterized by year-round ice or snow
Open water	Lakes and ponds

### Data Analysis

We tested for multicollinearity (*r*>0.7) of den-site variables to justify inclusion in candidate models. We used mixed model analysis of variance to compare den site landscape characteristics among adult female, adult male, and juvenile bears for relationships with elevation and slope. We controlled for repeated measures of bears (*n*≥1 den per individual) and treated year as a random effect, with bear ID nested within year. We used Tukey's range test for multiple comparisons. We compared den site aspects using chi-square analysis. We set α = 0.10 *a priori* for all analyses, as we expected our explanatory variables to vary greatly [58]. Land cover of den sites was summarized for each sex/age class. We also summarized variables between juvenile males and juvenile females to account for possible bias between these classes.

We used maximum entropy to model probable denning habitat (Maxent 3.3.3a; [56], [59]). Maximum entropy is a machine learning method for modeling species distributions from presence-only data, in which correlates at known locations are compared to the same correlates at 10,000 random points in the study area. Maximum entropy minimizes relative entropy between known location data and random point data [60]. Resulting models assign a 0 to 100 value (0 to 100% probability of occurrence) to all pixels, ranking them by relative suitability. Because maximum entropy compares presence locations to random locations, absence locations are not needed for analysis [61], [62].

We created separate models for adult female, adult male, and juvenile den sites using all variable combinations and each model was evaluated using receiver operating characteristic (ROC) plots. The ROC plots represent a model's ability to predict den locations and absences by plotting sensitivity against 1 – specificity [56]. We used the AUC statistic to select the most accurate model. Area under curve values range from 0.5 to 1.0, with 0.5 indicating no greater fit than expected by chance and 1.0 indicating perfect model fit [56]. We calculated standard errors for resulting AUC values by specifying that Maxent randomly set aside 30% of the den sites as test data. Maxent then used the remaining 70% of den sites as training data to fit a model, testing model fit using the test data. One problem with the AUC approach is that AUC values may be greatest for models with many variables even if some of those variables have negligible influence [56]. To account for this, we used a critical ratio test [63] to compare global models to the best 1–3 variable models for each sex/age class to see if improvement from additional variables was significant at α = 0.10. We then calculated Spearman rank correlation coefficients between competing models, and related the resulting coefficients to the table by Hanley and McNeil (1983) [64] to obtain adjusted correlation coefficients (*r*). These adjusted correlation coefficients were included in a critical ratio test [63]:
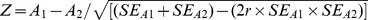
where *A*_1_ is the AUC value for the highest-ranked model, *A*_2_ is the AUC value for a lower-ranked model, and *SE* is the standard error for each respective model. We developed thresholds for probability of use by maximizing sensitivity and minimizing specificity and converted these results to a binary response of presence or absence [56]. Using the most parsimonious models, we mapped denning habitats of adult female, adult male, and juvenile grizzly bears.

## Results

From 1990–1998, we located 142 adult female, 36 adult male, and 35 juvenile (20 male, 15 female) den sites. Den-site elevation and slope were similar between juvenile males (elevation: 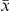 = 1,309 m, *SE* = 102; slope: 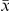 = 20.2°, *SE* = 2.2) and juvenile females (elevation: 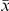 = 1,332 m, *SE* = 109; slope: 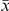 = 19.4°, *SE* = 3.6). Den-site elevation varied by gender/age class (F_2,63_ = 2.49, *P* = 0.091), with adult females denning at higher elevations than adult males (T_58_ = 2.22, *P* = 0.075). Juveniles denned at elevations similar to adult females (T_66_ = 1.02, *P* = 0.567) and adult males (T_66_ = 1.23, *P* = 0.443). Den-site slope also varied by sex/age class (F_2,84_ = 4.57, *P* = 0.013), with adult females denning on steeper slopes than adult males (T_62_ = 2.97, *P* = 0.011). Den-site slope of juveniles was similar to adult females (T_111_ = 1.45, *P* = 0.319) and adult males (T_105_ = 1.31, *P* = 0.391) (Table 2).

**Table 2:** Comparisons among grizzly bear den locations and habitat correlates for adult female (*n* = 142), adult male (*n* = 36), and juveniles (*n* = 35), Denali National Park and Preserve, Alaska, USA 1990–1998.

	Adult Female		Adult Male		Juvenile	
Variable	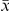 a	SE	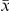 a	SE	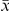 a	SE
Elevation (m)	1,412A	52	1,209B	76	1,329AB	66
Slope (°)	21.9A	1.1	15.6B	1.9	18.9AB	1.9

a Means not sharing a letter within rows differed significantly (*P*<0.10).

Bears showed non-random selection for aspect (χ^2^_8_ = 15.96, *P* = 0.043) with adult females and juveniles using east and south-facing aspects and adult males using east and west-facing aspects more than expected (Table 3). Both juvenile males and juvenile females selected east and south-facing aspects. Dwarf shrub and sparse vegetation were the two primary land covers of den sites for all sex/age classes. However, percentage of dens in each land cover varied, with adult female dens relatively equally distributed (45% dwarf shrub, 36% sparse vegetation), adult male dens primarily in the dwarf shrub class (58% dwarf shrub, 17% sparse vegetation), and juvenile dens primarily in the sparse vegetation land cover (55% sparse vegetation, 27% dwarf shrub) (Table 3). Sparse vegetation was the most common land cover class for both juvenile males and juvenile females.

**Table 3:** Number of grizzly bear den sites by aspect and covertype for adult females (*n* = 142), adult males (*n* = 36), and juveniles (*n* = 35), Denali National Park and Preserve, Alaska, USA 1990–1998.

Variable	Class	Adult Female	Adult Male	Juvenile
Aspect	North	22	5	4
	East	43	15	10
	South	43	5	12
	West	34	11	7
	Flat	0	0	2
Covertype	Open woodland spruce	1	3	1
	Broadleaf/mixed forest	0	0	1
	Alder/willow shrub	4	1	0
	Closed low birch shrub	0	0	1
	Low shrub/birch/willow/sedge	6	4	2
	Dwarf shrub	64	21	9
	Sparse vegetation	51	6	18
	Snow/ice	16	1	3

For maximum entropy modeling, we found no correlation between any variables for adult female or juvenile den sites (*r*≤0.70). Elevation and land cover was correlated for adult males (*r* = 0.72), thus, we did not run models containing elevation and land cover.

**Figure 1:**
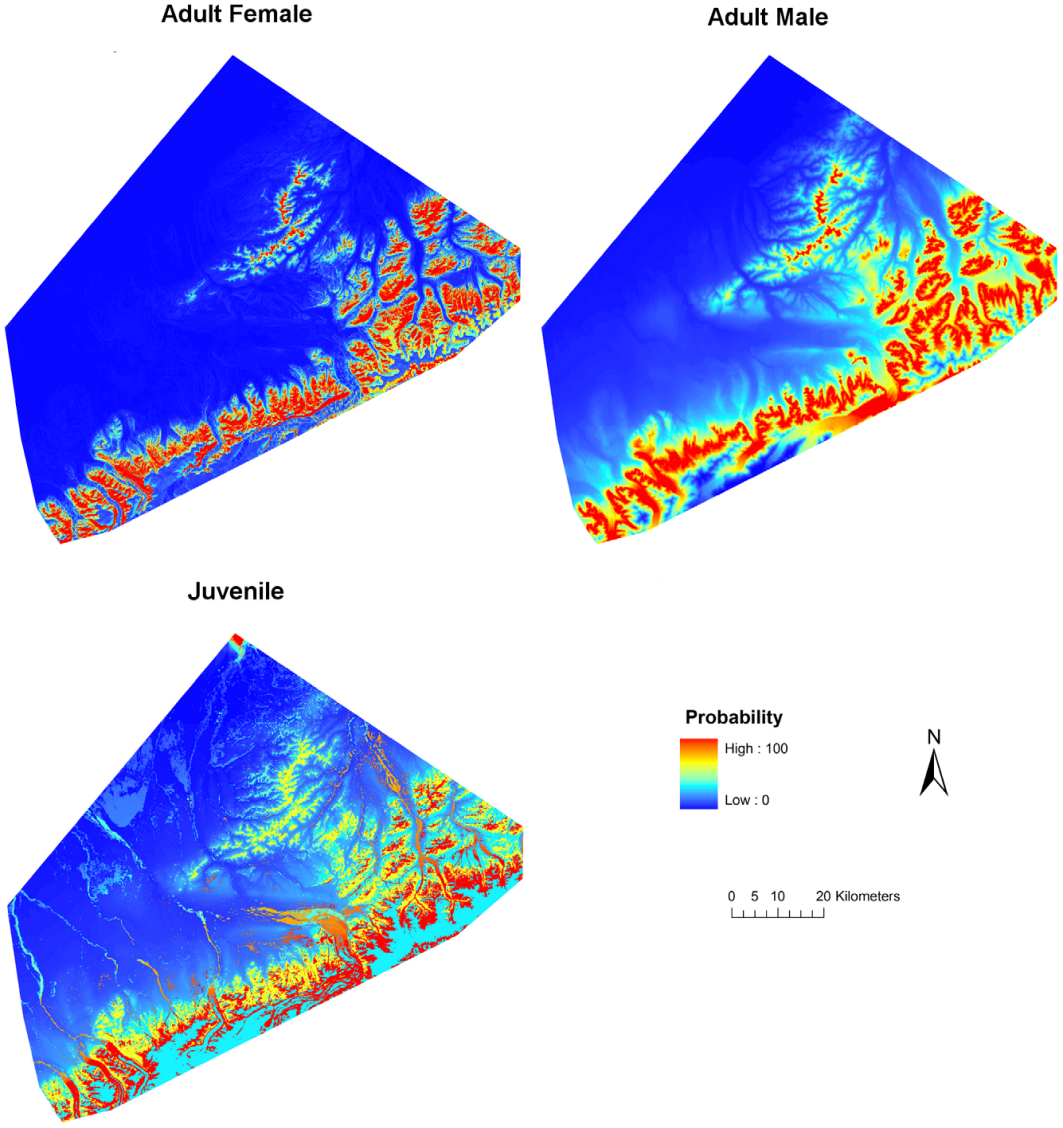
Probability of adult female, adult male, and juvenile grizzly bear denning habitat, Denali National Park and Preserve, Alaska, USA 1990–1998.

**Figure 2:**
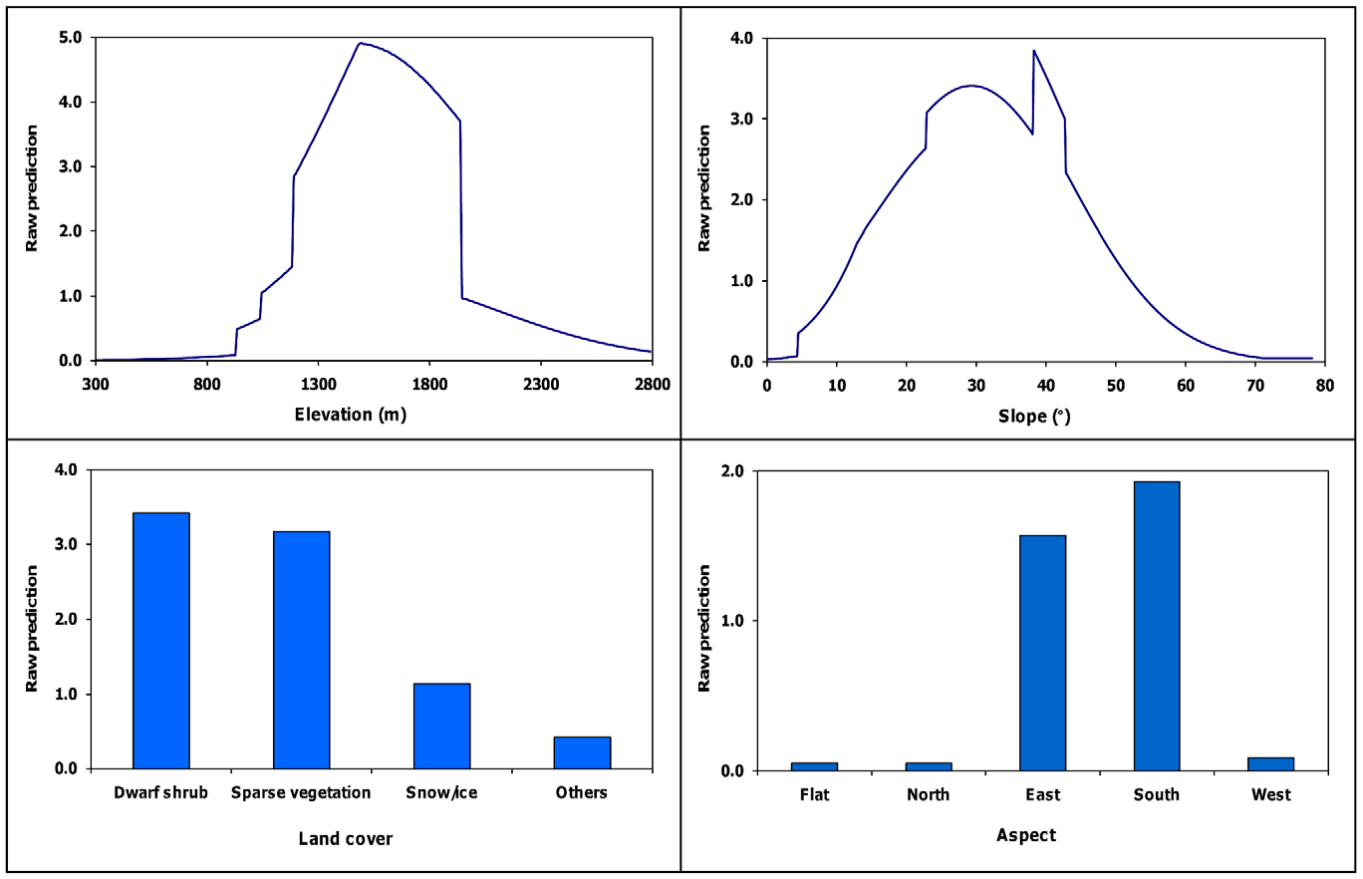
Adult female den-site selection model. Relationships between the exponential contribution of slope, elevation, land cover, and aspect to the raw prediction score and the observed value for 142 adult female grizzly bear den sites, Denali National Park and Preserve, Alaska, USA 1990–1998.

Best models for predicting den site use differed among adult females, adult males, and juveniles (Table 4). Based on AUC values and classification efficiency, the global model was most parsimonious for adult females (Fig. 1). This model was influenced most by elevation (contribution = 79.3%), followed by slope (12.7%), land cover (5.6%), and aspect (2.5%). Probability of den use increased with increasing elevation from 925 to 1,523 m, gradually decreased to 1,937 meters, and declined sharply thereafter (Fig. 2). Probability of den use also generally increased with increasing slope to 39°, declining thereafter. Den use was associated with east or south-facing aspects, and dwarf shrub/sparse vegetation land covers.

**Table 4:** Candidate maximum entropy models for adult female, adult male, and juvenile grizzly bear dens, Denali National Park and Preserve, Alaska, USA 1990–1998.

Age/Sex Class	Model^a^	AUC	SE	*Z*	*P*	Threshold	Class %
Adult Female	ESVA	0.926	0.002			19.900	83
	ESV	0.923	0.002	2.957	0.003	20.334	83
	EVA	0.920	0.002	5.624	<0.001	21.666	83
	ESA	0.919	0.002	2.673	0.008	21.118	83
	EV	0.916	0.002	9.373	<0.001	19.426	81
	SVA	0.910	0.002	11.153	<0.001	29.190	83
							
Adult Male	E	0.880	0.013			48.141	80
	EA	0.854	0.015	2.707	0.007	41.686	80
	ES	0.851	0.013	1.562	0.118	40.990	80
	ESA	0.840	0.014	4.050	<0.001	30.542	70
	SVA	0.838	0.022	1.841	0.066	30.647	80
	VA	0.831	0.027	1.849	0.065	33.814	80
							
Juvenile	EV	0.841	0.011			20.152	70
	SVA	0.824	0.012	1.893	0.058	18.824	80
	ESA	0.823	0.013	1.067	0.286	31.640	70
	EVA	0.823	0.013	1.071	0.284	19.478	80
	SV	0.823	0.015	1.963	0.049	18.778	70
	ESVA	0.819	0.012	3.088	0.002	14.922	70

a Model abbreviations: E = elevation, S = slope, V = land cover, A = aspect.

**Figure 3:**
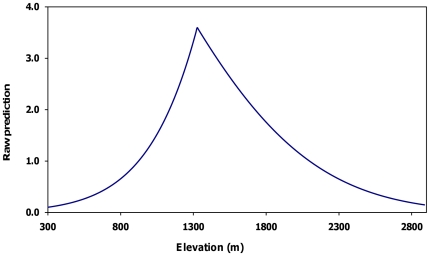
Adult male den-site selection model. Relationship between the exponential contribution of elevation to the raw prediction score and the observed value for 36 adult male grizzly bear den sites, Denali National Park and Preserve, Alaska, USA 1990–1998.

**Figure 4:**
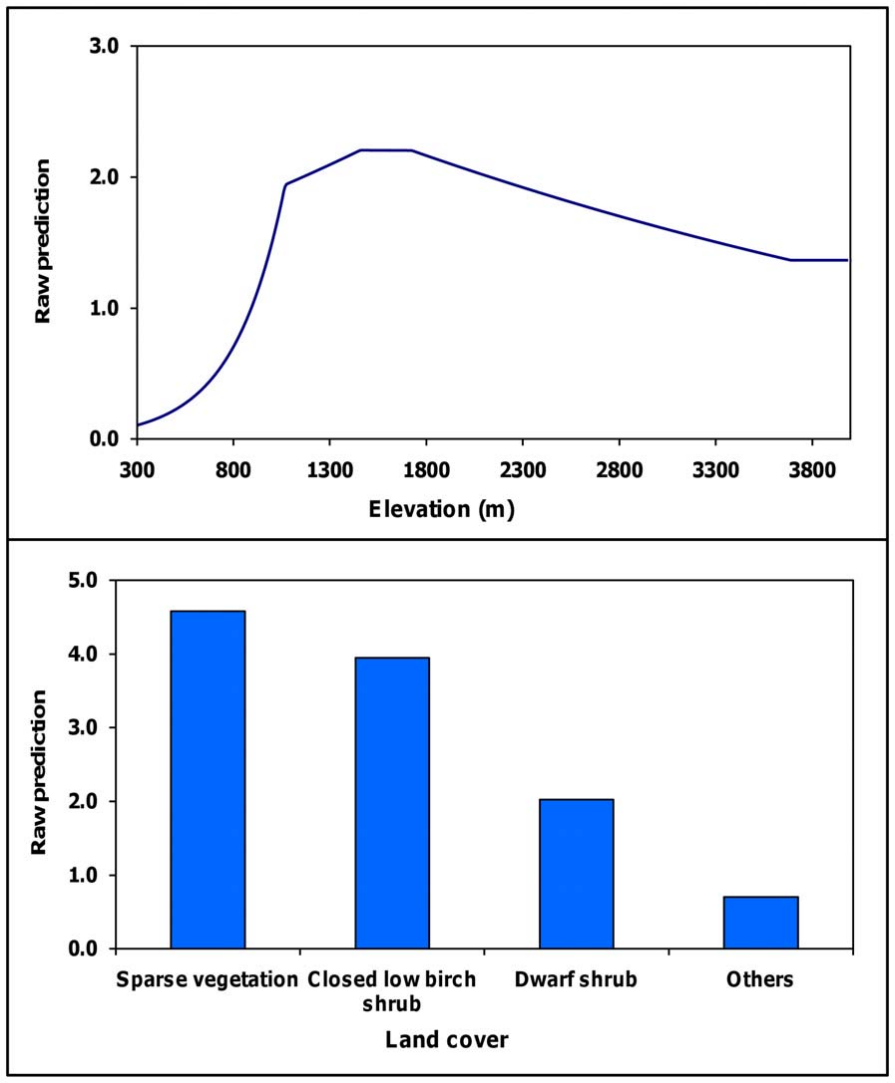
Juvenile den-site selection model. Relationship between the exponential contribution of land cover and elevation to the raw prediction score and the observed value for 35 juvenile grizzly bear den sites, Denali National Park and Preserve, Alaska, USA 1990–1998.

We selected the model containing elevation for adult males. Probability of den use increased with increasing elevation from 300 to 1,334 m and declined for areas >1,334 m (Figs. 1, 3). We selected the model containing land cover (contribution = 90.6%) and elevation (9.4%) for juveniles (Figs. 1, 4). Probability of den use was greater in areas with sparse vegetation, closed low birch shrub, and dwarf shrub land covers. There was a comparatively wide range of elevations associated with juvenile den use, with probability of use increasing with increasing elevation from 300 to 1,500 m, followed by a gradual decline.

## Discussion

### Risk and Den-Site Selection

Predation risk did not appear to influence juvenile den-site selection. Juveniles selected a wide range of elevations that did not differ from those selected by adult males. Thus, juvenile den-site selection did not appear to follow the ideal despotic distribution model. While it is possible that adult males selectively kill juvenile males to eliminate potential competitors and increase breeding opportunities, small sample size for juvenile den locations (*n* = 35, 20 male, 15 female) precluded rigorous testing between juvenile females and juvenile males. Thus, we were unable to test whether this potential source of predation risk affected juvenile male den location. However, our results supported the ideal despotic distribution model and the sex hypothesis of sexual segregation for adult females, in that den-site selection differed between adult females and adult males, with maximum probability of den use for adult females at higher elevations than for adult males. The sex hypothesis of sexual segregation, coupled with adult females denning for longer periods of time than adult males [47]–[49], is the most likely explanation for observed adult female den use. As predation risk is similar for juveniles and adult females (the risk to the individual), we suggest that observed sexual segregation is a consequence of adult females avoiding adult males to reduce risk of infanticide (the added risk of losing their reproductive investment).

Although the mechanism for male den-site selection is unknown, we suggest the range of elevations selected by males was related to food availability at den emergence. Grizzly bears in Denali National Park and Preserve emerge from dens around May [50], coinciding with caribou parturition on their calving ground in our study area [65]. Den site elevation use of adult male grizzly bears overlapped extensively with the elevation range of the caribou calving ground, with highest number of newborn calves generally occurring from 900 to 1,500 m [65]. Mortality of caribou calves due to bear predation averaged 22% annually [50]. We suggest this concentrated and available food source was exploited by adult male grizzly bears to increase lean body mass following den emergence (e.g., Belant et al. 2006 [29]), leading to improved condition. Improved body condition (e.g., larger size) provides males with greater breeding opportunities in many species [26], [66]–[68].

In addition to denning at higher elevations, adult females entering dens earlier and emerging later may also be in response to infanticide risk [47]–[49]. Adult females, particularly those with young, should delay denning to maximize foraging opportunities before winter as percentage body fat in fall influences proportion of lean body mass lost during hibernation, and therefore animal condition [44]. However, we suggest that by moving to high elevation den locations early, adult females are further reducing the risk of infanticide, by moving through adult male denning areas before occupation by adult males. Likewise, it would be energetically advantageous for adult females to leave dens earlier to forage, because females with young lose more body mass than lone bears during hibernation [44]. However, this would necessitate adult females moving through high concentrations of adult males in denning areas. Further, the most readily available food in our study area in early spring was caribou calves or carcasses of animals that died in winter. These concentrated food sources are likely to attract multiple bears, including adult males, similar to concentrations of salmon [12], [69]. These food resources are therefore risky for adult females with young [12], [70]–[72]. Consequently, adult females may remain in high elevation dens to conserve energy, where longer snow cover increases thermal insulation and reduces energy loss, and wait for adult males to disperse from den areas and more dispersed food (e.g., herbaceous vegetation) to become available. We suggest that predation risk alone does not strongly influence den-site selection in grizzly bears. However, the added risk of infanticide appears to influence adult female den-site selection and contributes to spatial segregation between adult females and adult males.

**Figure 5:**
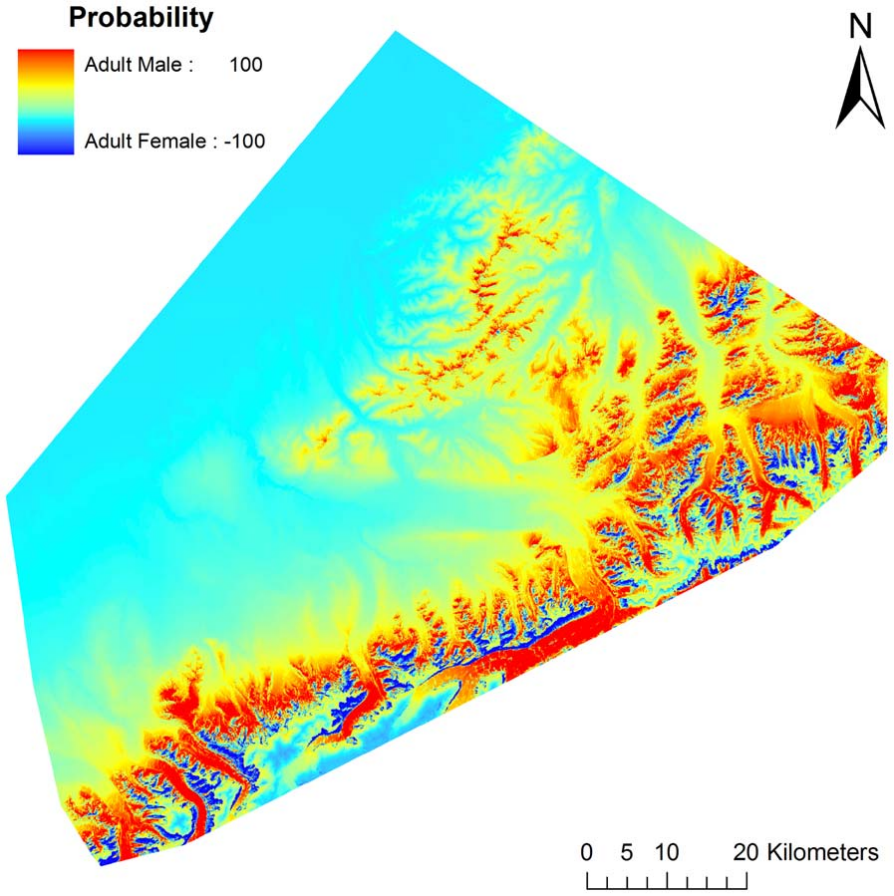
Shift in probability of suitable den locations between adult female and adult male grizzly bears, Denali National Park and Preserve, Alaska, USA 1990–1998. Cooler colors represent areas more suitable for adult females and warmer colors represent areas more suitable for adult males.

Though not addressed in our hypotheses, our results suggest adult females may further spatially segregate by occupying steeper slopes than adult males [45]. This difference, however, may also be an artifact of the observed elevation gradient among sex/age classes as higher elevations often exhibit steeper slopes. Though elevation and slope differed between adult females and adult males, it is important to note there was considerable overlap. We suggest that while adult females attempted to sexually segregate, they were constrained by topographic (i.e., elevation) and structural (e.g., slope) features. These requirements likely limited how high and steep adult females could den, as very steep slopes are structurally unstable and the highest elevations in the study area have permafrost or little topsoil conducive to den excavation. Adult females in this study exhibited greatest selection for a narrow band of habitat near ridges (Fig. 5). Consequently, although segregation of adult females from adult males was observed, segregation may have been constrained by landscape features.

### Den-Site Characteristics

Elevation was an important indicator of den sites for adult females and adult males, with high probability of use associated with mid-elevation portions of the study area. These elevations likely provided good insulative snow cover while remaining free of permafrost [30]. Although elevation was a predictor in the juvenile model, it only contributed 9.4% to model fit. The difference in contribution of elevation between juveniles and adult bears may be a consequence of inexperience. Resource use of juveniles often differs from adults and has been attributed to naïveté [73], [74], which may in part explain high observed variability in juvenile den elevation, resulting in low explanatory power.

Slope was moderately important for predicting adult female denning habitat, contributing 12.7% to model fit. Strongest selection was for slopes between 22–39°. These values are within the range reported in other studies, and likely were selected in part for structural stability and drainage properties [30], [75]–[78].

Land cover was the best predictor of juvenile denning habitat (contribution = 90.6%). Sparse vegetation and closed low birch shrub were the most probable cover types, followed by dwarf shrub. These cover types are indicative of higher elevation sites generally chosen for den sites. Land cover was also present in our adult female model but only contributed 5.6% to model fit. Sparse vegetation, dwarf shrub, and snow cover types were probable denning habitat. These cover types are consistent with den elevations, and we believe were an artifact of selection for elevation.

### Conclusions

Risk from conspecifics influences resource selection in many species [10]–[23]. Adult male grizzly bears selected den sites in areas with abundant, high quality food available at den emergence (i.e., caribou calves [65]). We suggest that adult males selected these areas to improve individual fitness and increase breeding opportunities [26], [28]. That adult male and juvenile den-site selection was similar suggests predation risk was not a strong indicator of den-site selection by juveniles. However, risk of infanticide appeared to influence adult female den-site selection, with adult females selecting higher elevations and steeper slopes than adult males. We suggest sexual segregation is an important component of grizzly bear denning ecology, providing a mechanism by which adult females avoid infanticidal males. As adult male grizzly bears are the dominant sex/age group and adult female denning behavior appears suboptimal from an energetic perspective, observed sexual segregation supports the ideal despotic distribution model. While empirical evidence supporting sexual segregation to reduce infanticide is limited [18], [20], a growing body of literature suggests it occurs frequently across numerous taxa [15], [18], [21], [23], [24], [46].
